# Diagnostic value of ^18^F-FDG PET/CT versus contrast-enhanced MRI for venous tumour thrombus and venous bland thrombus in renal cell carcinoma

**DOI:** 10.1038/s41598-021-04541-9

**Published:** 2022-01-12

**Authors:** An-hui Zhu, Xiao-yan Hou, Shuai Tian, Wei-fang Zhang

**Affiliations:** 1 Department of Nuclear Medicine, Peking University Third Hospital, 49 North Garden Rd, Haidian District, Beijing, 100191 People’s Republic of China; 2 Department of Radiology, Peking University Third Hospital, Beijing, 100191 China

**Keywords:** Cancer imaging, Renal cancer

## Abstract

The purpose of this study was to compare the ability of ^18^F-FDG PET/CT and contrast-enhanced MRI (CEMRI) to detect and grade venous tumour thrombus (VTT) and venous bland thrombus (VBT) in RCC and assess invasion of the venous wall by VTT. The PET/CT and CEMRI data of 41 patients with RCC were retrieved. The difference in maximum standardized uptake value (SUVmax) between VTT and VBT was analysed. According to their pathological diagnosis, the patients were divided into those with and without venous wall invasion. The PET/CT and CEMRI features, including the SUVmax of the primary lesion and VTT, maximum venous diameter, complete occlusion of the vein by VTT, and VTT morphology, were compared between the two groups. All 41 patients had VTT, and eleven of the 41 patients had VBT. The mean SUVmax of the VTT (6.33 ± 4. 68, n = 41) was significantly higher than that of the VBT (1.37 ± 0.26, n = 11; *P* < 0.001). Ten of the 11 cases of VBT were correctly diagnosed by ^18^F-FDG PET/CT, and all 11 were diagnosed by CEMRI. Both ^18^F-FDG PET/CT and CEMRI can effectively detect VTT and distinguish VTT from VBT. ^18^F-FDG PET/CT is less effective in grading VTT than CEMRI. Complete venous occlusion by VTT indicates venous wall invasion.

## Introduction

Renal cell carcinoma (RCC) reportedly invades the venous system and forms venous tumour thrombus (VTT) in 4–36% of patients^1^. Postoperatively, the survival of patients with RCC who undergo venous thrombectomy is significantly worse than that of patients with localized RCC, especially those with level III-IV VTT, as these patients have higher perioperative risk and mortality^2^.

If no IVC wall is present, removal of the VTT is sufficient. In the case of IVC wall invasion, it is necessary to perform segmental resection or even prosthetic replacement to prevent postoperative recurrence or venous insufficiency. Therefore, preoperative assessments to identify VTT with venous wall invasion is crucial for surgical planning in patients with RCC^3^. Recurrence and mortality rates are high in these patients, especially in those with advanced disease and invasion of the venous wall by VTT^4^. In the present study, imaging methods such as computed tomography (CT), magnetic resonance imaging (MRI), and ultrasonography were commonly used to evaluate VTT in patients with renal cell carcinoma. Imaging-based staging and restaging of RCC is commonly performed using ^18^F-FDG PET/CT^5–8^. Hallscheidt et al.^9^ and Adams et al.^10^ used contrast-enhanced MRI (CEMRI) to identify invasion of the venous wall from VTT in RCC due to its intrinsic contrast superiority. To date, there has been no systematic comparison between ^18^F-FDG PET/CT and CEMRI in their ability to grade VTT and detect venous bland thrombus (VBT) and venous wall invasion by VTT.

The aims of this study were as follows: (1) to compare the ability of ^18^F-FDG PET/CT and CEMRI to detect VTT and VBT in RCC; (2) to compare the ability of ^18^F-FDG PET/CT and CEMRI to grade VTT; and (3) to evaluate the ability of both ^18^F-FDG PET/CT and CEMRI to assess venous wall invasion by VTT.

## Results

### Assessment of patients based on ^18^F-FDG PET/CT and CEMRI

The 41 RCCs were clear cell carcinoma (n = 34) or type 2 papillary renal carcinoma (n = 7). Both PET/CT and CEMRI detected all 41 primary renal tumours, which had a mean maximum diameter of 8.7 ± 3.5 (range 3.0–15.0) cm. Primary renal carcinoma showed a renal mass with varying degrees of increased FDG uptake. The mean SUVmax for the primary lesions was 7.82 ± 7.09 (range 2.32–36.93). On CEMRI, all 34 clear RCCs were enhanced, and the 7 type 2 papillary renal carcinomas showed uneven or low enhancement compared to the clear RCCs.

Both PET/CT and CEMRI correctly detected VTT in all 41 cases and showed good consistency in the grading of VTT (κ = 0.964). PET/CT correctly graded 40 cases and underestimated 1 case, whereas CEMRI correctly graded all 41 cases (the relationship between the radiological grade of VTT determined by PET/CT and MRI and the clinical grade is shown in Table 1). Eleven of the 41 patients had VBT. Ten of 11 cases of VBT were correctly diagnosed by PET/CT, and all 11 correctly diagnosed by CEMRI. There was excellent interobserver agreement for determining VBT between PET/CT and CEMRI, with a kappa coefficient of 0.936.

**Table 1:** Relationship between VTT level on PET/CT and CEMRI and clinical stage.

Clinical level	n	Imaging level	
		PET/CT	CEMRI
0	4	4	4
I	4	4	4
II	20	21	20
III	6	6	6
IV	7	6	7
n	41	41	41

*CEMRI* contrast-enhanced magnetic resonance imaging, *PET/CT* positron emission tomography/computed tomography.

### Assessment of VTT and VBT based on ^18^F-FDG PET/CT and CEMRI

**Figure 1:**
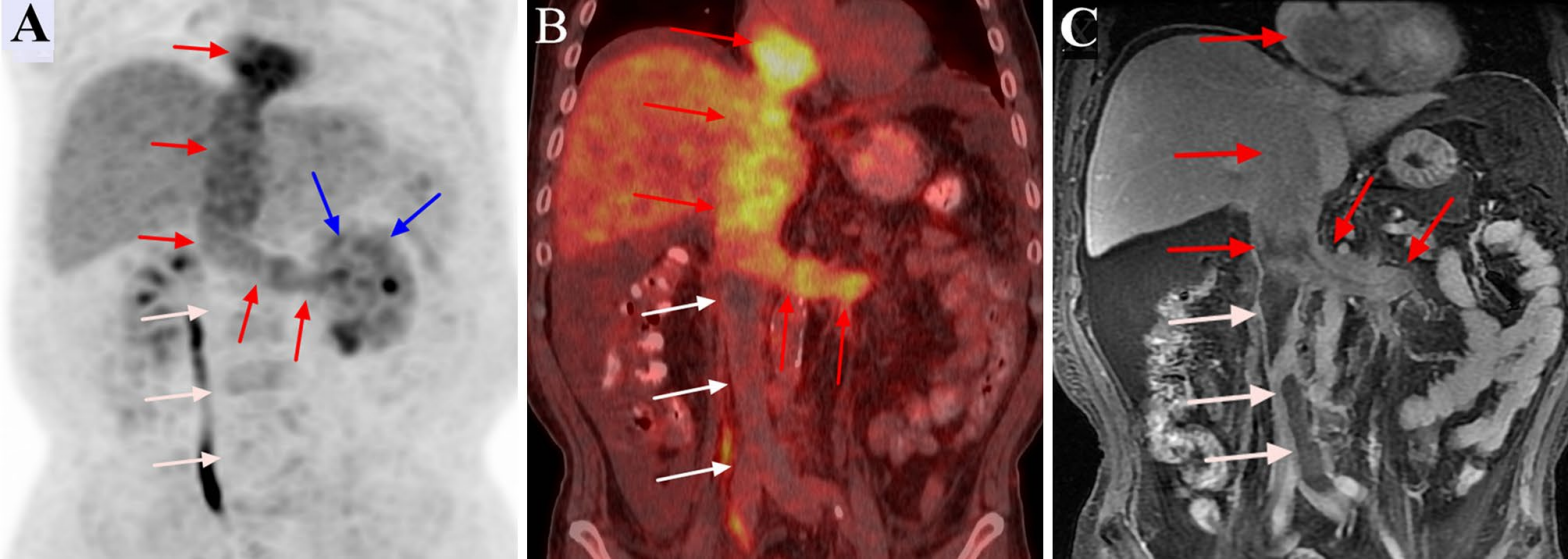
^18^F-FDG positron emission tomography (PET)/computed tomography (CT) and contrast-enhanced MRI (CEMRI) scans of a 67-year-old man who had clear cell carcinoma of the left kidney with a level IV venous tumour thrombus (VTT) and venous bland thrombus (VBT). The VTT had invaded the venous wall and was graded as level IV by both PET/CT and CEMRI. The site of diffusely increased FDG uptake (SUVmax 5.3) on the PET maximum intensity projection image is the primary tumour (blue arrow) (**a**). The site of pathologically increased FDG uptake (SUVmax 5.7) on the PET and PET/CT images from the left renal vein to the inferior vena cava is the VTT (red arrow) (**a**, **b**). The focal site of decreased FDG uptake (SUVmax 1.2) at the inferior vena cava is the VBT (white arrow) (**a**, **b**). The VTT with a regular shape shows heterogeneous enhancement and completely occludes the inferior vena cava lumen on coronal CEMRI (red arrow) (**c**), while the VBT shows no enhancement (white arrow) (**c**).

**Figure 2:**
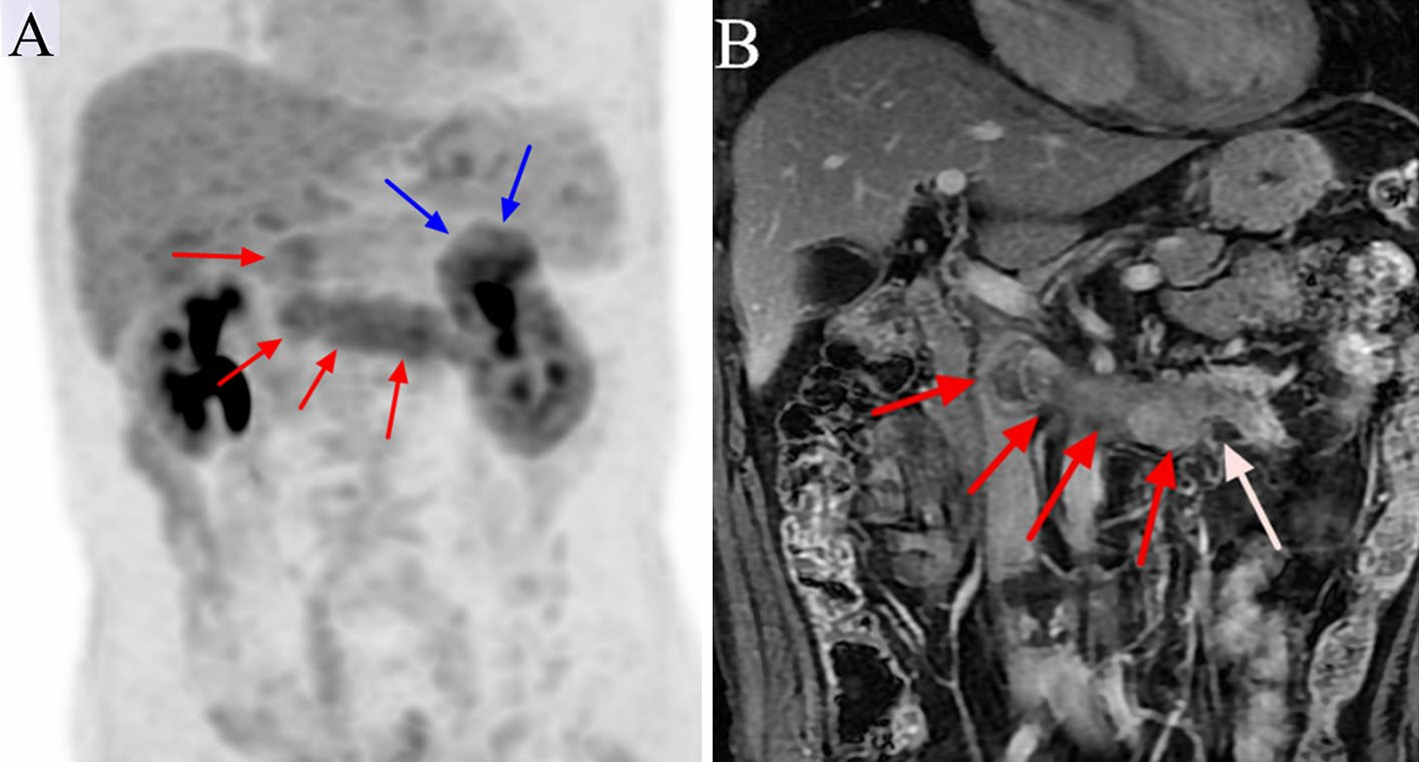
^18^F-FDG PET/CT and contrast-enhanced MRI (CEMRI) scans of a 75-year-old man with clear cell carcinoma of the left kidney associated with a level II venous tumour thrombus (VTT) and small venous bland thrombus (VBT). The VTT did not invade the venous wall and was graded as level II by PET/CT and CEMRI. The site of focally increased FDG uptake (SUVmax 5.8) on the PET maximum intensity projection image is the primary tumour (blue arrow) (**a**). A PET maximum intensity projection image (**a**) shows a site of pathologically increased FDG uptake (SUVmax 4.4) from the left renal vein to the inferior vena cava that is the VTT (red arrow). The VTT has a regular shape, is homogeneously enhanced, and completely occludes the inferior vena cava lumen on coronal CEMRI (red arrow) (**b**). The small VBT shows no enhancement on coronal CEMRI (white arrow) (**b**).

**Figure 3:**
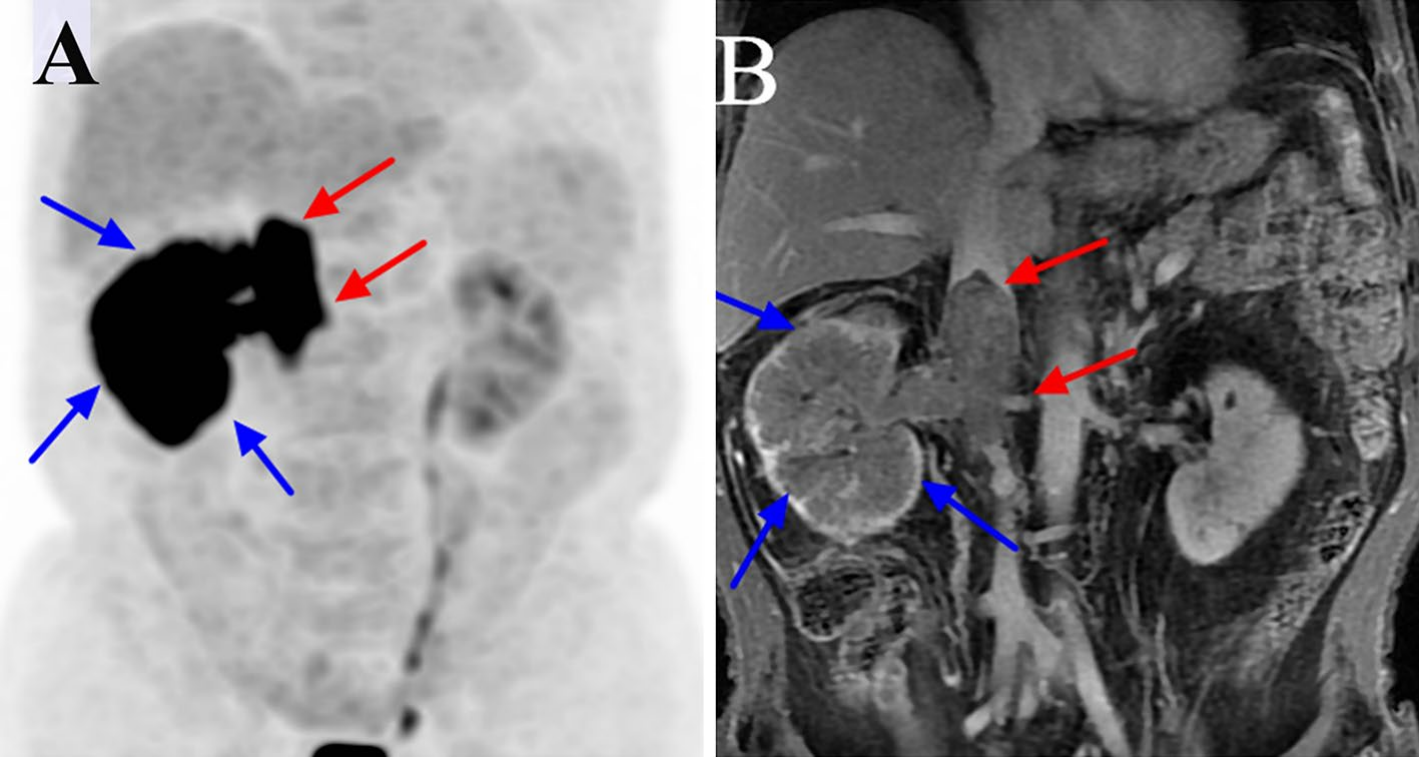
^18^F-FDG PET/CT and contrast-enhanced MRI (CEMRI) scans of a 77-year-old man with papillary cell carcinoma of the right kidney associated with a level II venous tumour thrombus (VTT). The VTT had invaded the venous wall and was graded as level II by PET/CT and CEMRI. The site of diffusely increased FDG uptake (SUVmax 9.6) on the PET maximum intensity projection image is the primary tumour (blue arrow) (**a**). The site of pathologically increased FDG uptake (SUVmax 12.5) from the right renal vein to the inferior vena cava is the VTT (red arrow) (**a**). The VTT had a regular shape, was enhanced and completely occluded the inferior vena cava lumen on coronal CEMRI (red arrow) (**b**), and the primary tumour showed diffuse heterogeneous enhancement (blue arrow) (**b**).

**Figure 4:**
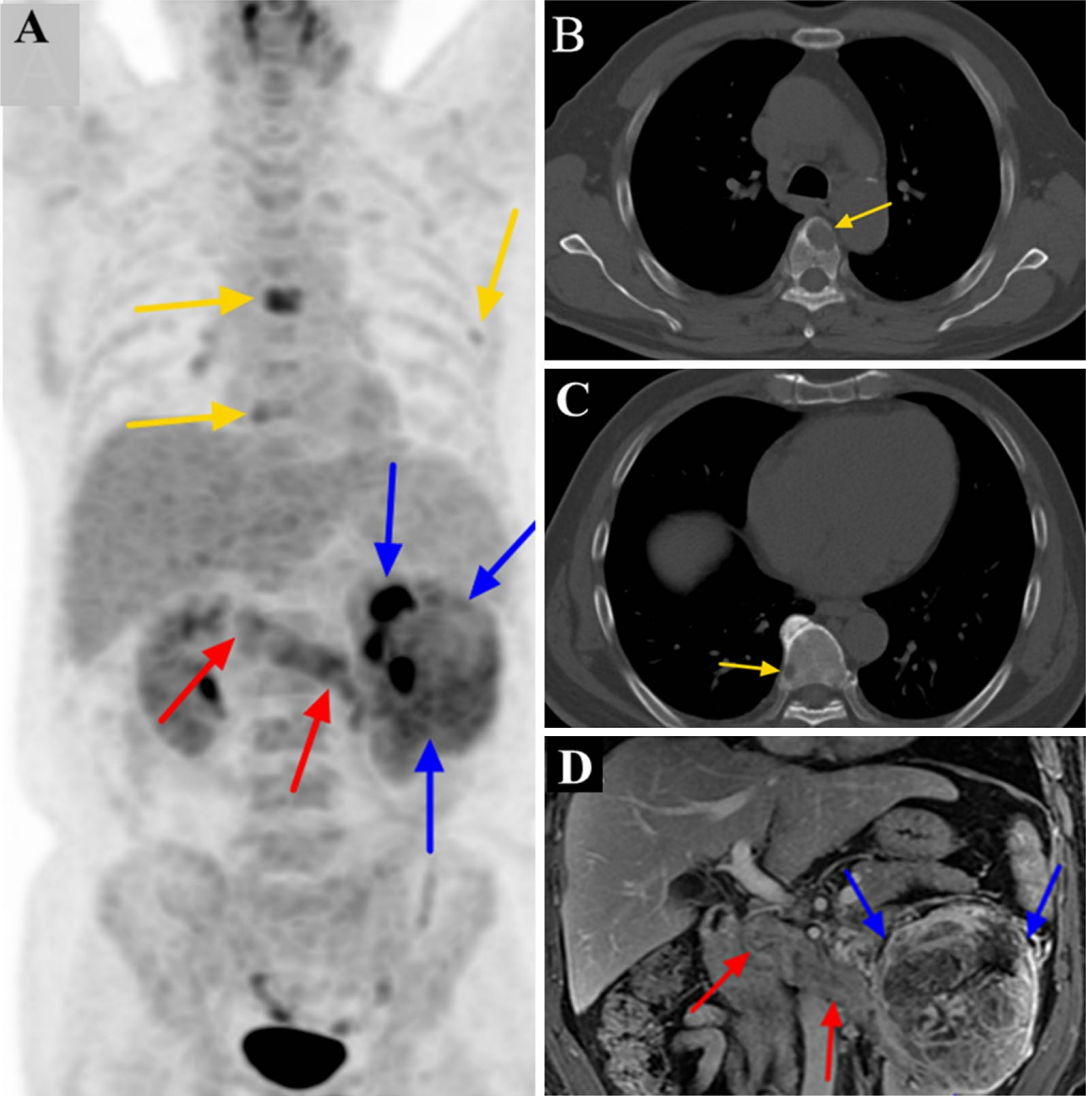
^18^F-FDG PET/CT and contrast-enhanced MRI (CEMRI) scans of a 59-year-old man with clear cell carcinoma of the left kidney associated with a level II venous tumour thrombus (VTT). The VTT invaded the venous wall and was graded as level II by both PET/CT and CEMRI. The site of diffusely increased FDG uptake (SUVmax 4.3) on a PET maximum intensity projection image is the primary tumour (blue arrow) (**a**). The site of pathologically increased FDG uptake (SUVmax 4.5) on the PET image from the left renal vein to the inferior vena cava is the VTT (red arrow) (**a**). Some bone metastases in the thoracic vertebrae and a left rib showed increased focal FDG uptake (yellow arrow) (**a**) with lytic lesions on selected axial CT slices (yellow arrow) (**b**,**c**). The VTT did not show homogeneous enhancement and completely occluded the lumen of the inferior vena cava on coronal CEMRI (red arrow) (**d**). The primary tumour showed heterogeneous enhancement (blue arrow) (**d**).

**Figure 5:**
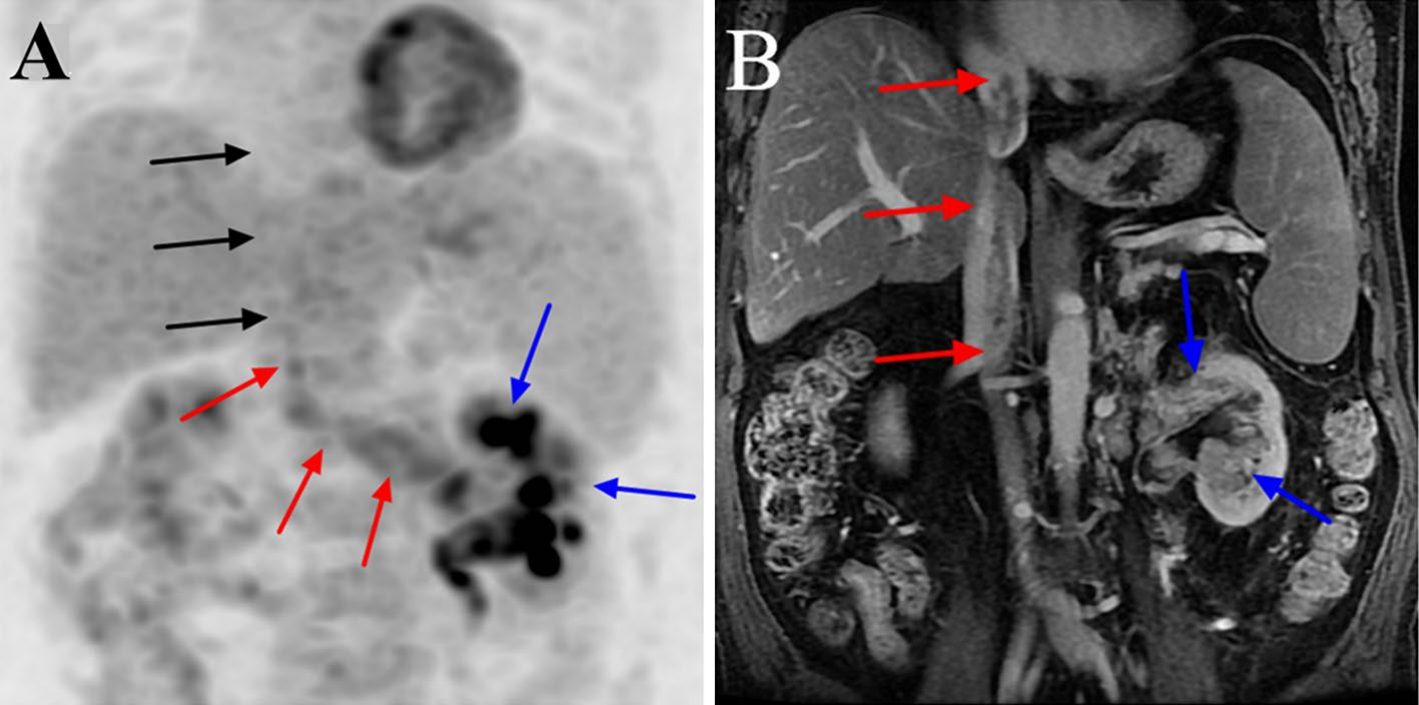
^18^F-FDG PET/CT and contrast-enhanced MRI (CEMRI) scans of a 62-year-old man with clear cell carcinoma of the left kidney associated with a level IV venous tumour thrombus (VTT). The VTT did not invade the venous wall and was graded as level II by PET/CT and level IV by CEMRI. The site of diffusely increased FDG uptake (SUVmax 6.3) on a PET maximum intensity projection image is the primary tumour (blue arrow) (**a**). The VTT showed pathologically increased FDG uptake (SUVmax 3.4) from the left renal vein to the inferior vena cava below the hepatic vein (red arrow) (**a**). However, there was no increased FDG uptake (SUVmax 2.0) from the hepatic vein to the inferior vena cava above the diaphragm (black arrow) (**a**). Coronal CEMRI showed that the VTT had an irregular shape, was heterogeneously enhanced, and partially occluded the vein lumen (red arrow) (**b**). The primary tumour also showed heterogeneous enhancement (blue arrow) (**a**).

On PET/CT, the VTT had higher FDG uptake than the abdominal aorta in 40 cases (Figs. 1, 2, 3, 4); in 1 case, the uptake close to the proximal renal hilum was higher than that of the abdominal aorta, while the uptake close to the proximal outflow tract was closer to that of the abdominal aorta (Fig. 5). The mean SUVmax of the VTT was 6.33 ± 4.68 (range 1.93–25.96). In ten of the 11 patients, the VBT had lower FDG uptake than the abdominal aorta; the remaining case was misdiagnosed (Fig. 2). The mean SUVmax of the VBT was 1.37 ± 0.26 (range 0.85–1.82). The mean SUVmax of the VTT was significantly higher than that of the VBT (*P* < 0.001).

On MRI, the flow void signal for VTT disappeared on plain T2WI, and heterogeneous enhancement appeared on enhanced T1WI scans in all 41 cases (Figs. 1, 2, 3, 4, 5). All 11 cases of VBT were correctly diagnosed by MRI (Figs. 1 and 2). The flow void signal for VBT disappeared on plain T2WI, and no enhancement appeared on enhanced T1WI scans in all 11 cases.

### Assessment of venous wall invasion by VTT based on ^18^F-FDG PET/CT and CEMRI

In Group 1, the mean SUVmax measurements for the primary lesion and VTT and the maximum diameter of the venous lumen were 9.58 ± 9.25, 6.75 ± 5.89, and 3.12 ± 0.75 cm, respectively; the corresponding values in Group 2 were 6.16 ± 3.72, 5.92 ± 3.17, and 3.42 ± 0.89 cm.

The association between complete occlusion of the vein by VTT on CEMRI and IVC invasion was significant (*P* < 0.001). Complete and incomplete occlusion of the vein by VTT was seen in 17 cases and 3 cases, respectively, in Groups 1 and 5 and in 16 cases in Group 2. This feature was associated with a significantly higher probability of IVC invasion with a sensitivity of 85%, specificity of 76.2%, positive predictive value of 77.3%, and negative predictive value of 84.2%.

The maximum diameter of the venous lumen, VTT morphology, SUVmax of the primary lesion, and SUVmax of VTT were not associated with a significantly higher probability of IVC wall invasion (*P* > 0.05).

## Discussion

PET/CT can simultaneously evaluate the anatomy of a lesion and provide relevant metabolic information and is used widely in the clinical staging and postoperative restaging of RCC. Many studies have shown that the diagnostic results of primary RCC obtained by ^18^F-FDG-PET/CT vary according to tumour pathology and nuclear differentiation grade^6,7^. There are varying reports on the diagnostic sensitivity of ^18^F-FDG-PET/CT for primary RCC lesions^5,11^. CEMRI provides superior soft-tissue resolution and can show the extent of the primary tumour and VTT involvement. This imaging modality is also suitable for patients with iodine contrast allergies or impaired renal function. CEMRI can help to predict the various pathological types of RCC by showing the enhancement mode and apparent diffusion coefficient values^12,13^. One study showed that the sensitivity of CEMRI for detecting primary tumours varied according to tumour size; when the primary tumour was ≤ 4 cm, the sensitivity was 88.1%^14^. In the present study, both PET/CT and CEMRI had a detection rate of 100%, which are higher than those previously reported and can be explained by the large mean maximum diameter of the primary lesions. Thirty-one of the 41 primary lesions were high-grade RCC, in which the FDG uptake was consistently higher than that in the normal renal background, enabling easier detection. Ten of the 41 primary lesions were low-grade RCC, in which the FDG uptake was low and similar to that of the normal renal background. These 10 low-grade tumours were detected due to their large size. However, there were some potential shortcomings of the Fuhrmann grading system used in the present study: the FDG uptake in low-grade RCC may be close to or lower than that in the renal parenchyma and is not easily recognized; and low-grade RCC that does not cause changes to the renal contour or density may be missed due to their small size.

Image quality is essential in patients with RCC and VTT or VBT^15^. Previous research has shown that ^18^F-FDG PET/CT can help to distinguish VBT from VTT because FDG uptake is absent in VBT but present in VTT^8^. The investigators of that study reported that CEMRI afforded good soft-tissue resolution and showed the site and extent of VTT accurately. PET/CT graded VTT correctly in 40 patients but underestimated the grade in 1 case. CEMRI detected all 41 cases of VTT. The features of VTT on MRI were the disappearance of the flow void signal on plain T2WI and heterogeneous enhancement in the venous lumen on enhanced T1WI. There were 11 cases of VBT that were distributed in or located at the distal end of a VTT, in which the FDG uptake was lower than that in the adjacent VTT. PET/CT detected 10/11 cases of VBT and missed a small VBT that was obscured by a VTT. However, CEMRI detected all 11 cases of VBT correctly. This finding indicates that both PET/CT and CEMRI could detect VTT and VBT effectively but that CEMRI is more effective for the classification of VTT and detection of very small bland thrombi.

In another study that included 48 patients with RCC complicated by VTT (including 26 with venous wall invasion), complete occlusion of the IVC lumen or vessel breach on CEMRI could reliably indicate invasion of the IVC wall with a sensitivity of 92.3%, a specificity of 86.4%, a positive predictive value of 88.9%, and a negative predictive value of 90.5%^10^. The mean diameter of the venous lumen harbouring the VTT was significantly larger in patients with wall invasion than in those without wall invasion. In our study, 22 of 41 patients had complete occlusion of the IVC lumen on CEMRI, and 17 of 22 patients had invasion the venous wall. Complete occlusion of the IVC lumen was utilized to predict invasion of the IVC wall, with a sensitivity of 85%, a specificity of 76.2%, a positive predictive value of 77.3%, and a negative predictive value of 84.2%. In 23 out of 41 patients with complete occlusion of the IVC lumen, 13 patients underwent open radical nephrectomy, and 9 patients underwent retroperitoneal laparoscopic radical nephrectomy. The maximum diameter of the venous lumen, VTT morphology, SUVmax of the primary lesion, and SUVmax of the VTT were not associated with a higher probability of IVC wall invasion. However, there has not been a systematic report on the ability of PET/CT and CEMRI to detect wall invasion.

Adams et al.^16^ tested the potential of unenhanced cardiac- and respiratory-motion-corrected three-dimensional steady-state free precession (3D-SSFP) magnetic resonance imaging for the assessment of VTT in 18 patients with clear-cell renal cell carcinoma (cRCC) and compared this technique to standard contrast-enhanced (CE)-MRI and CE-computed tomography (CT). The study indicated that 3D-SSFP can achieve an accurate assessment of IVC thrombi in cRCC patients without the need for contrast medium and is superior to standard MRI and CT. There was 100% agreement between 3D-SSFP and the surgical findings, 83.3% agreement between CEMRI and the surgical findings, and 71.4% agreement between CECT and the surgical findings regarding the level of the IVC thrombus. Alayed et al.^17^ evaluated MRI in the diagnosis of IVC wall invasion by VTT in 24 patients with RCC. The study indicated that patients with VTT with invasion had larger renal vein (28 ± 8 vs. 15 ± 6 mm; P = 0.031) and IVC (41 ± 9 vs. 19 ± 6 mm; P = 0.003) diameters, greater craniocaudal extent (87 ± 34 vs. 51 ± 31 mm; P = 0.0239), and greater volume (77.4 ± 57.6 vs. 17.7 ± 17.4 cm^3^; P = 0.003) than those with did VTT without invasion. Previous research suggested that tumour size, histological subtype, and thrombus morphology were independent predictors of overall and cancer-specific survival in patients with RCC and IVC thrombi^18^. Choi et al.^18^ analysed the impact of morphological features of VTT on the overall survival and cancer-specific survival of 156 RCC patients. The study showed that thrombus morphology was an independent predictor of overall survival and cancer-specific survival in RCC patients. In _p_N_O_ /_X_ M_O_ patients with round tumours with smooth margins, the presence of VTT significantly affected cancer-specific survival (P = 0.003) compared to patients with tumours with irregular, sharp or friable margins. Another study demonstrated that venous wall invasion by a tumour thrombus would impact the risk of tumour recurrence and survival^4^. Therefore, it is important to develop appropriate surgical plans based on preoperative morphological features.

In the present study, 23 of 41 patients underwent open radical nephrectomy for invasion of the venous wall by VTT (n = 9), a primary tumour with a diameter of 10–16 cm (n = 9, including 4 cases of venous wall invasion), and a primary tumour invading the renal fascia (n = 5). Eighteen of 41 patients underwent retroperitoneal laparoscopic radical nephrectomy for invasion of the venous wall by VTT (n = 7) and a primary tumour without invasion of the renal fascia (n = 11). The eighteen of 41 patients who underwent retroperitoneal laparoscopic radical nephrectomy included 3 cases of level 0, 4 cases of level I and 11 cases of level II VTT. In this study, 4 patients with level 0 VTT and 4 patients with level I VTT underwent open radical nephrectomy (n = 1) and retroperitoneal laparoscopic radical nephrectomy (n = 7). One of 4 patients with level 0 VTT underwent open radical nephrectomy due to the presence of a large primary tumour with a diameter of 16 cm and spleen invasion, and another patient with level I VTT developed bilateral lung metastasis. For patients with level 0 and I VTT, PET/CT can be performed to determine the presence of metastasis and complicated VBT and assess the relationship between the primary lesion and the adjacent organs, which is helpful for creating a surgical plan. For patients with level 0 and I VTT without distant metastasis or primary invasion of the adjacent organs, retroperitoneal laparoscopic radical nephrectomy can be performed; for patients with level 0 and I VTT with distant metastasis or primary invasion of adjacent organs, adjuvant chemotherapy or targeted therapy before surgery or open radical nephrectomy is recommended. One of the 41 patients underwent segmental resection of the vena cava after intraoperative transoesophageal echocardiography (TOE), which showed that the thrombus was in the right atrium and attached to the wall of the IVC. Intraoperative TOE can be used to further characterize the mobility and consistency of the thrombus^19^. A recent study demonstrated that TOE provided new diagnostic information and impacted surgical management in all 15 patients with a level IV thrombus and that the diagnostic yield of intraoperative TOE was higher in patients with greater tumour thrombus extension into the vena cava^20^.

The main limitation of this study was its small sample size. Only 41 patients who underwent both ^18^F-FDG PET/CT and CEMRI were included. Therefore, although the results of this study were statistically significant, further confirmatory research with a larger sample size is needed.

In conclusion, both ^18^F-FDG PET/CT and CEMRI can effectively detect VTT and distinguish VTT from VBT. Complete venous occlusion by VTT indicates venous wall invasion. However, compared with CEMRI, PET/CT is more helpful for evaluating distant organ metastasis in RCC. Surgeons need to choose the best individual diagnostic and therapeutic option.

## Methods

### Patients

The study was approved by the Peking University Third Hospital Medical Science Research Ethics Committee. Informed consent was obtained from all patients who were included in the study. All methods were performed by following the relevant guidelines and regulations in the study. The database was reviewed to identify all patients with biopsy-proven malignancy. We identified 41 patients (7 women, 34 men; mean age 63.7 ± 11.1 [range 46–82] years) with a diagnosis of RCC with VTT in whom integrated whole-body ^18^F-FDG PET/CT and CEMRI were performed preoperatively (at a median 3 [range 1–7] days) between February 2012 and November 2020.

**Figure 6:**
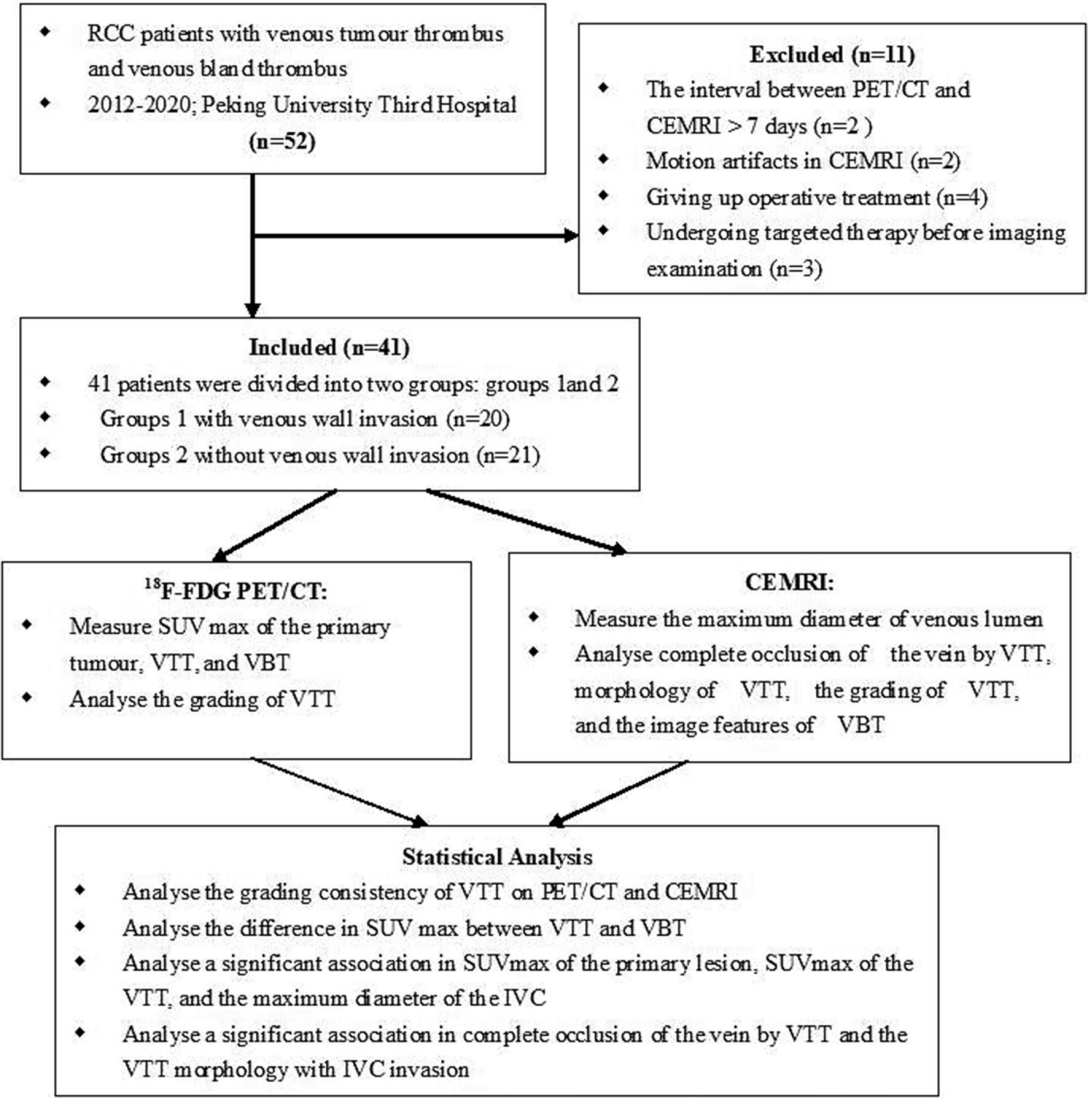
The study design of 18F-FDG PET/CT and contrast-enhanced MRI for venous tumour thrombus and venous bland thrombus in the patient population.

The pathological diagnosis was made according to the 2004 World Health Organization classification standard for RCC^21^, and nuclear grading was performed using the Fuhrman system^22^. The classification of VTT was based on the Mayo Clinic standard^23^. Clinical staging was performed according to the 7th edition of the American Joint Committee on Cancer criteria for RCC. Twenty-three of the 41 patients underwent open radical nephrectomy, and 18 underwent retroperitoneal laparoscopic radical nephrectomy; segmental resection of the vena cava was performed in 9 cases. One of the 41 patients underwent TOE during surgery, and the remaining 40 did not because TOE was not performed routinely for these patients. The pathological diagnoses were type 2 papillary RCC (n = 7) and clear cell carcinoma (n = 34). The Fuhrman grade was high in 31 cases and low in 10. The clinical VTT grade was level 0 in 4 cases, level I in 4, level II in 20, level III in 6, and level IV in 7. The VTT had invaded the venous wall in 20 cases, and a VBT had formed in 11 cases. One of the 41 patients had multiple VTTs in a pulmonary artery, 4 had adrenal metastasis, and 13 had distant organ metastasis. Among 7 patients with level IV tumour thrombus, the VTT invaded the IVC wall in 3 cases, two patients presented with oedema of both lower extremities, two patients presented with haematuria, and two patients were asymptomatic. An overview of the patient characteristics is provided in Table 2. Figure 6 shows the study design for 18F-FDG PET/CT and contrast-enhanced MRI evaluations for venous tumour thrombus and venous bland thrombus in this patient cohort.

**Table 2:** Characteristics of the study population.

Parameter	Value
Patients, n	41
Men/women, n/n	34/7
Mean age at surgery (range; standard deviation)	63.7 (46–82; 11.1)
Involvement of the right kidney, n	26
**Thrombus level, n (%)**	
0	4 (9.8)
I	4 (9.8)
II	20 (48.8)
III	6 (14.5)
IV	7 (17.1)
**Fuhrman grade, n (%)**	
1	1 (2.4)
2	9 (22.0)
3	24 (58.5)
4	7 (17.1)
**TNM classification, n (%)**	
T3a	7 (17.1)
T3b	6 (14.5)
T3c	20 (48.8)
T4	8 (19.6)
Clear cell carcinoma, n (%)	34 (82.9)
Papillary carcinoma, n (%)	7 (17.1)
**Surgical technique**	
Open radical nephrectomy + segmental resection of vena cava	9
Open radical nephrectomy + vena cava thrombectomy	14
Retroperitoneal laparoscopic radical nephrectomy + vena cava thrombectomy	18
Presence of preoperative metastases, n (%)	13 (31.7)

### Imaging protocol

#### ^18^F-FDG PET/CT

All patients underwent imaging with a 52-ring Biograph Integrated PET/CT system (Siemens Healthineers, Erlangen, Germany). ^18^F-FDG was provided by the Institute of Isotope Research at the China Institute of Atomic Energy. After fasting for more than 6 h, each patient received 5.55 MBq/kg (0.15 mCi/kg) of ^18^F-FDG intravenously. Routine PET/CT was performed 60 min later. The CT scans extended from the skull base to the upper thigh with a matrix size of 512 × 512. PET images were acquired with a matrix size of 168 × 168 in 5–7 bed positions (2–2.5 min per bed) and then reconstructed by OSEM. The images were subsequently fused and evaluated using a MedEx PET/CT image and information system.

#### Contrast-enhanced MRI

CEMRI was performed using a 3.0-T Discovery MR750w unit (GE Healthcare, Chicago, IL, USA). The scanning range was from the upper edge of the diaphragm to the upper edge of the iliac bone. Before the intravenous administration of a contrast agent, all patients underwent routine imaging of the kidneys, which included axial and coronal T2-weighted single-shot fast spin-echo without fat saturation sequence, an axial three-dimensional T1-weighted in-phase sequence, and an opposed-phase gradient-recalled echo sequence (liver acquisition with volume acceleration [LAVA]). All patients underwent routine axial and coronal three-dimensional T1-weighted fat-suppression LAVA imaging with an intravenous administration of a bolus of gadopentetate (Beijing BeiLu Pharmaceutical Co., Ltd, Beijing, China) at a dose of 0.1 ml/kg injected at a rate of 1.5–2.0 ml/s, followed by a 20-ml saline flush. Contrast-enhanced images were acquired in the corticomedullary and nephrographic phases using an automatic bolus triggering technique. The nephrographic phase was initiated 30–40 s after the corticomedullary phase (see Table 3 for MRI parameters).

**Table 3:** Details of parameters used in magnetic resonance imaging sequences.

Type of acquisition	T2 SSFSE^a ^coronal	T2 SSFS^a^ axial	T1 opposed-phase LAVA^b^ axial	T1 in-phase LAVA^b^ axial	T1 FS LAVA^c^ coronal	T1 FS LAVA^c^ axial
TR (ms)	1074	1400	4	4	4	4
TE (ms)	70	70	1.2	2.4	1.8	1.8
FOV	400 × 400	400 × 400	360 × 360	360 × 360	360 × 360	360 × 360
Matrix size	288 × 192	288 × 192	264 × 256	264 × 256	272 × 192	264 × 256
Slice thickness (mm)	6	6	3	3	1.5	3
Pixel band width (Hz/pixel)	244	244	558	558	558	558
Acquisition mode	2D	2D	3D	3D	3D	3D
Flip angle (°)	90	90	10	10	10	10
Voxel size	1.4 × 2.1 × 6	1.4 × 2.1 × 6	1.4 × 1.4 × 3	1.4 × 1.4 × 3	1.4 × 1.4 × 1.5	1.4 × 1.4 × 3

*FOV* field of view, *TE* echo time, *TR* repetition time.

^a^Single-shot fast spin echo sequence.

^b^Gradient-recalled echo sequence (liver acquisition with volume acceleration sequence).

^c^Fat-suppression gradient-recalled echo sequence (liver acquisition with volume acceleration sequence).

### Image analysis

The PET/CT and CEMRI features were rereviewed by two blinded radiologists with 8 and 11 years of experience and two blinded nuclear medicine physicians with 9 and 12 years of experience.

The PET/CT features were analysed visually and semiquantitatively on a Siemens SyngoMMWP VE40A workstation. The ^18^F-FDG uptake, location, and morphological changes in low-dose CT lesions were determined visually. For the diagnosis of renal carcinoma, concordant mild, moderate or intense ^18^F-FDG uptake in a CT-visualized anatomical lesion was required. VTT was diagnosed when the FDG uptake in the venous lumen on PET was higher than that in the background of the abdominal aorta at the same level, whereas VBT was diagnosed when the FDG uptake in the venous lumen on PET images was approximately equal or lower than that in the adjacent vein or background of the abdominal aorta at the same level^8,24^. The maximum standardized uptake value (SUVmax) of ^18^F-FDG was measured semiquantitatively as the highest uptake in the kidney and venous lesions.

All of the CEMRI images were analysed using PACS workstations (Centricity Radiology; GE Healthcare). A lesion with nonuniform enhancement was diagnosed as renal carcinoma on CEMRI. VTT was diagnosed if the flow void signal was observed on plain T2WI and if nonuniform enhancement was observed in the venous lumen on enhanced T1WI. A VBT was diagnosed if the flow void signal disappeared on plain T2WI and there was no enhancement in the venous lumen on enhanced T1WI.

The maximum diameter of the venous lumen was measured on axial CEMRI scans. Complete occlusion of the vein by VTT (defined as no passage of contrast within the vein) was observed on enhanced axial and coronal T1WI. The morphology of the VTT was analysed on enhanced coronal T1WI: if the contour was smooth and continuous, it was assumed to be regular; if the contour was uneven, it was assumed to be irregular. The VTT classification was based on the Mayo Clinic standard^6^.

### Data analysis

The imaging features of the primary tumour, VTT, and VBT observed on PET/CT and CEMRI were analysed. The VTT grades obtained on PET/CT and CEMRI were compared with those obtained clinically. The SUVmax of the primary tumour, VTT, and VBT were also recorded, as was the maximum diameter of the venous lumen in which the VTT was located. According to their pathological diagnosis, the patients were divided into those with and without venous wall invasion (Groups 1 and 2, respectively).

### Statistical analysis

Data are reported as the mean ± SD or total number (%). The interobserver agreement between PET/CT and CEMRI for the detection of VBT was quantified by the kappa statistic. The consistency of VTT grading by PET/CT and CEMRI was also quantified by the kappa statistic. The Mann–Whitney U test was used to analyse the difference in SUVmax values between VTT and VBT. The Mann–Whitney U test was used to assess whether the SUVmax of the primary lesion, SUVmax of the VTT, and maximum diameter of the venous lumen showed a significant association with IVC wall invasion. Pearson’s chi-square test was used to assess whether complete occlusion of the vein by VTT and VTT morphology showed a significant association with IVC wall invasion. All statistical analyses were performed using SPSS for Windows (version 18.0; IBM Corp., Armonk, NY, USA). A P value < 0.05 was considered statistically significant.

## Data Availability

The datasets generated during and/or analysed during the current study are available from the corresponding author on reasonable request.

## References

[1] Pouliot F, Shuch B, Larochelle JC, Pantuck A, Belldegrun AS (2010). Contemporary management of renal tumors with venous tumor thrombus. J. Urol..

[2] Tilki D (2014). Impact of histologic subtype on cancer-specific survival in patients with renal cell carcinoma and tumor thrombus. Eur. Urol..

[3] Calero A, Armstrong PA (2013). Renal cell carcinoma accompanied by venous invasion and inferior vena cava thrombus: Classification and operative strategies for the vascular surgeon. Semin. Vasc. Surg..

[4] Armstrong PA (2014). Outcomes after inferior vena cava thrombectomy and reconstruction for advanced renal cell carcinoma with tumor thrombus. J. Vasc. Surg. Venous Lymphat. Disord..

[5] Ozülker T, Ozülker F, Ozbek E, Ozpaçaci T (2011). A prospective diagnostic accuracy study of F-18 fluorodeoxyglucose-positron emission tomography/computed tomography in the evaluation of indeterminate renal masses. Nucl. Med. Commun..

[6] Fuccio C (2014). Restaging clear cell renal carcinoma with 18F-FDG PET/CT. Clin. Nucl. Med..

[7] Takahashi M (2015). Preoperative evaluation of renal cell carcinoma by using 18F-FDG PET/CT. Clin. Nucl. Med..

[8] Ravina M, Hess S, Chauhan MS, Jacob MJ, Alavi A (2014). Tumor thrombus: Ancillary findings on FDG PET/CT in an oncologic population. Clin. Nucl. Med..

[9] Hallscheidt P, Pomer S, Roeren T, Kauffmann GW, Staehler G (2000). Preoperative staging of renal cell carcinoma with caval thrombus: Is staging in MRI justified? Prospective histopathological correlated study. Urol. A..

[10] Adams LC (2018). Renal cell carcinoma with venous extension: Prediction of inferior vena cava wall invasion by MRI. Cancer Imaging.

[11] Park JW, Jo MK, Lee HM (2009). Significance of 18F-fluorodeoxyglucose positron-emission tomography/computed tomography for the postoperative surveillance of advanced renal cell carcinoma. BJU Int..

[12] Yamamoto A (2017). Differentiation of subtypes of renal cell carcinoma: Dynamic contrast-enhanced magnetic resonance imaging versus diffusion-weighted magnetic resonance imaging. Clin. Imaging..

[13] Mytsyk Y (2017). Renal cell carcinoma: Applicability of the apparent coefficient of the diffusion-weighted estimated by MRI for improving their differential diagnosis, histologic subtyping, and differentiation grade. Int. Urol. Nephrol..

[14] Kim JH (2016). Diagnostic accuracy of contrast-enhanced computed tomography and contrast enhanced magnetic resonance imaging of small renal masses in real practice: Sensitivity and specificity according to subjective radiologic interpretation. World J. Surg. Oncol..

[15] Woodruff DY (2013). The perioperative management of an inferior vena caval tumor thrombus in patients with renal cell carcinoma. Urol. Oncol..

[16] Adams LC (2018). Assessing venous thrombus in renal cell carcinoma: Preliminary results for unenhanced 3D-SSFP MRI. Clin. Radiol..

[17] Alayed A (2019). Diagnostic accuracy of MRI for detecting inferior vena cava wall invasion in renal cell carcinoma tumor thrombus using quantitative and subjective analysis. Am. J. Roentgenol..

[18] Choi DK (2017). Surgical treatment of renal cell carcinoma: Can morphological features of inferior vena cava tumor thrombus on computed tomography or magnetic resonance imaging be a prognostic factor?. Int. J. Urol..

[19] Psutka SP (2015). Clinical and radiographic predictors of the need for inferior vena cava resection during nephrectomy for patients with renal cell carcinoma and caval tumour thrombus. BJU Int..

[20] Kostibas MP (2017). Defining the role of intraoperative transesophageal echocardiography during radical nephrectomy with inferior vena cava tumor thrombectomy for renal cell carcinoma. Urology.

[21] Lopez-Beltran A, Scarpelli M, Montironi R, Kirkali Z (2006). 2004 WHO classification of the renal tumors of the adults. Eur. Urol..

[22] Fuhrman SA, Lasky LC, Limas C (1982). Prognostic significance of morphologic parameters in renal cell carcinoma. Am. J. Surg. Pathol..

[23] Blute ML, Leibovich BC, Lohse CM, Cheville JC, Zincke H (2004). The Mayo Clinic experience with surgical management, complications and outcome for patients with renal cell carcinoma and venous tumour thrombus. BJU Int..

[24] Davidson T, Goitein O, Avigdor A, Zwas ST, Goshen E (2009). 18F-FDG-PET/CT for the diagnosis of tumor thrombosis. Isr. Med. Assoc. J..

